# Supplementary figures and images for: Evaluation and comparison of methods for neuronal parameter optimization using the Neuroptimus software framework
Source: PLoS Comput Biol. 2024 Dec 23;20(12):e1012039. doi: 10.1371/journal.pcbi.1012039 (PMC11706405; doi:10.1371/journal.pcbi.1012039)

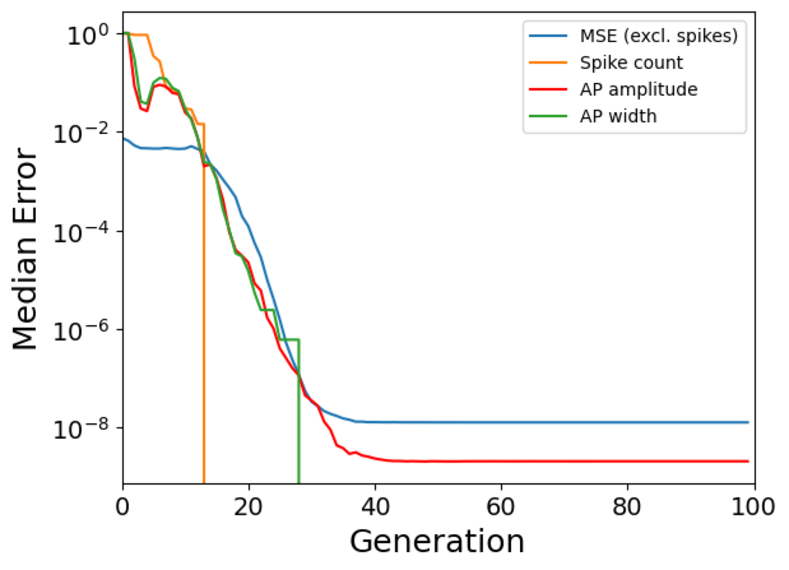

Supplement: S1 Fig — The curves show the median of 10 independent runs. Each generation corresponds to 100 model evaluations. (TIF) [file pcbi.1012039.s001.tif]

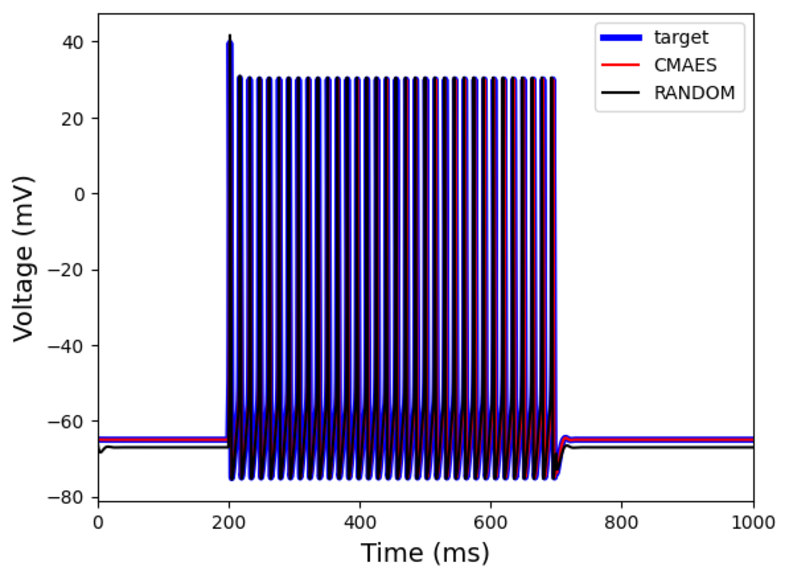

Supplement: S2 Fig — (TIF) [file pcbi.1012039.s002.tif]

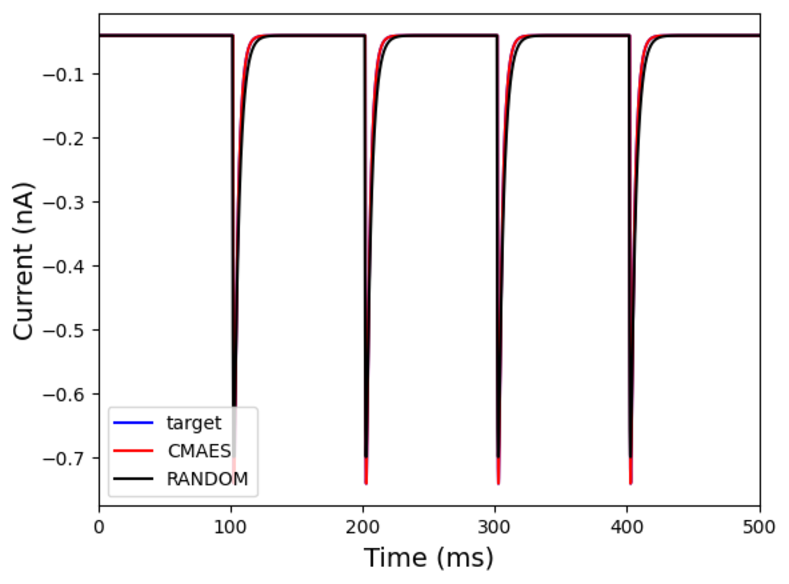

Supplement: S3 Fig — (TIF) [file pcbi.1012039.s003.tif]

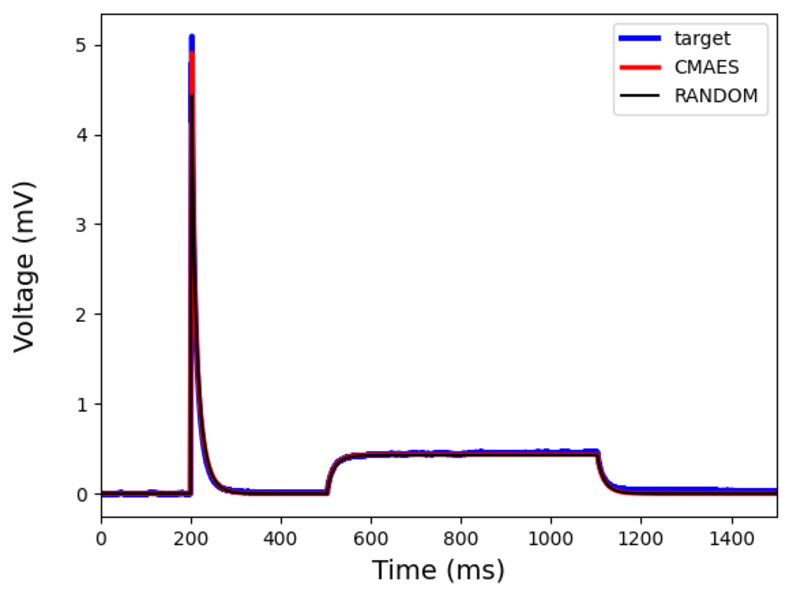

Supplement: S4 Fig — (TIF) [file pcbi.1012039.s004.tif]

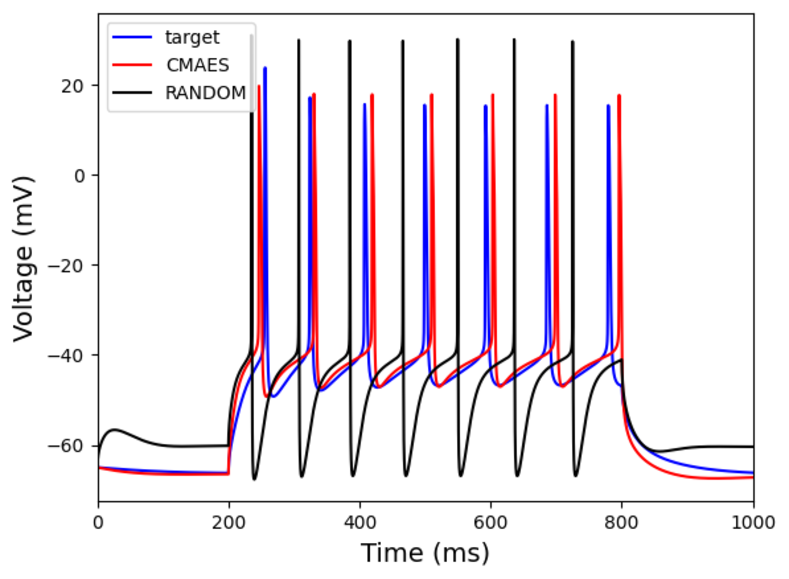

Supplement: S5 Fig — (TIF) [file pcbi.1012039.s005.tif]

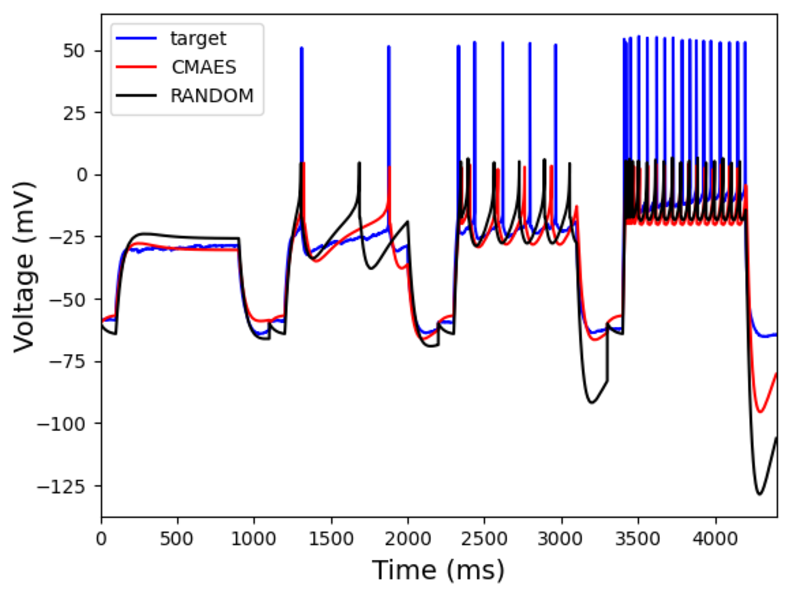

Supplement: S6 Fig — (TIF) [file pcbi.1012039.s006.tif]

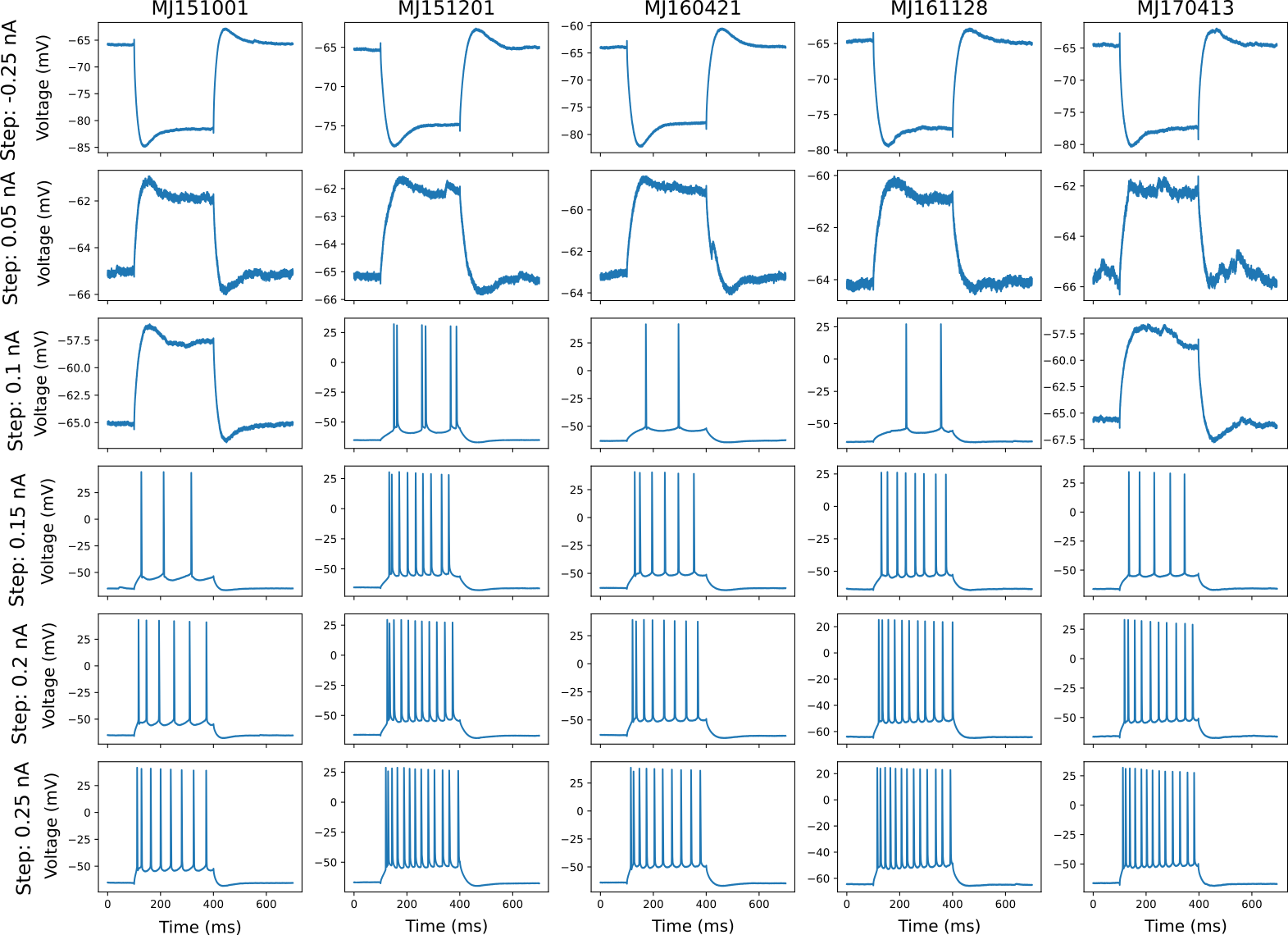

Supplement: S7 Fig — For each cell the current step protocol was repeated 3 times, and the figure shows the results of the first recordings from the cells. Features extracted from these experimental data (5 cells and 3 repeated recordings, which resulted in 15 recordings) were used as the target data in Use Case 6 (morphologically and biophysically detailed CA1 pyramidal cell model). (TIF) [file pcbi.1012039.s007.tif]

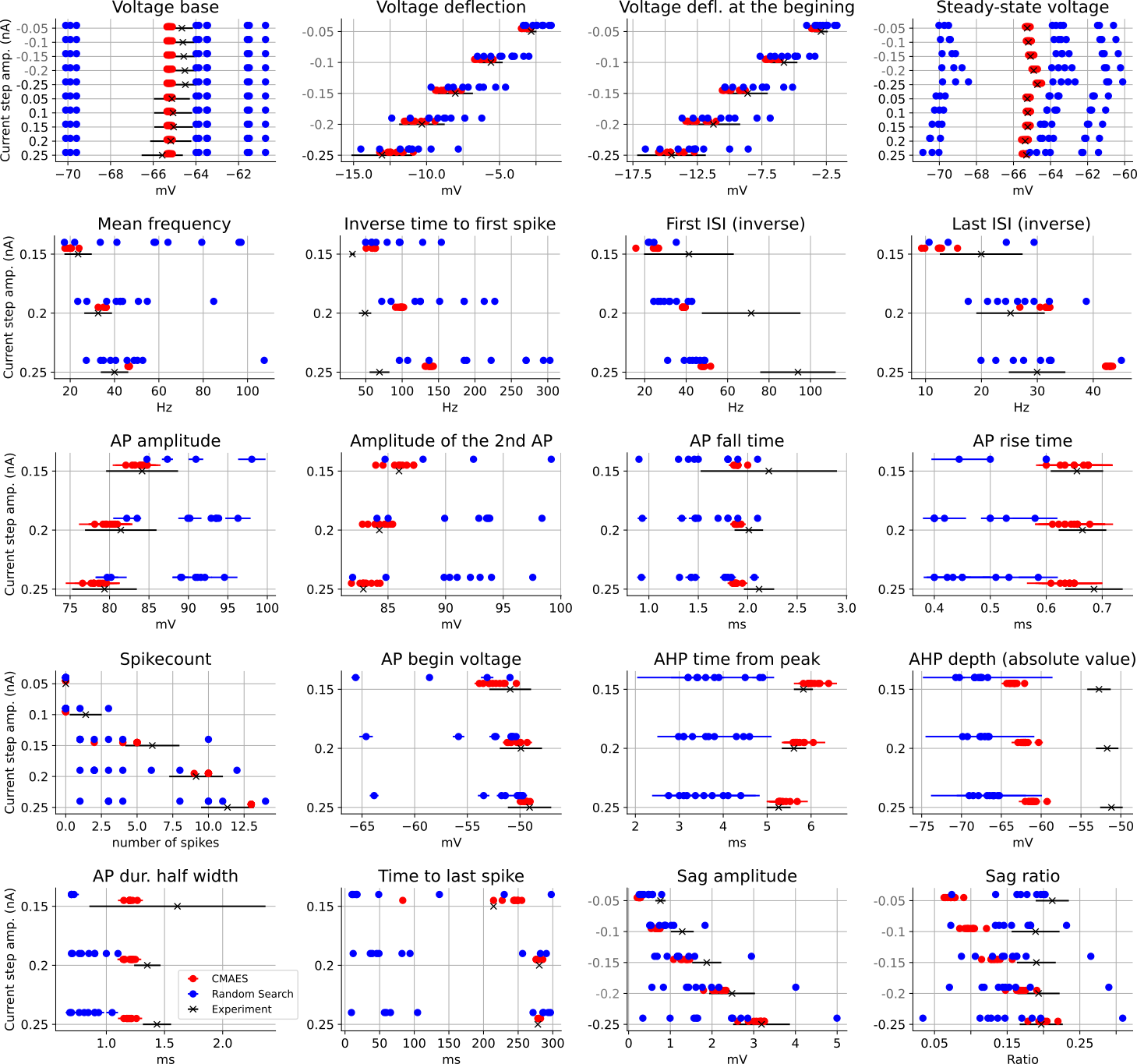

Supplement: S8 Fig — The experimental mean and standard deviation are represented by the black cross and error bar, while the red and blue dots correspond to the feature values in simulations of the best parameter combinations resulting from 10 independent runs of the CMAES and random search algorithms, respectively. Values along the vertical axis denote the amplitude of the current injection used. Note that the feature values from the CMAES solutions are typically closer to the experimental mean values, and have smaller variability, than those resulting from random search. (TIF) [file pcbi.1012039.s008.tif]

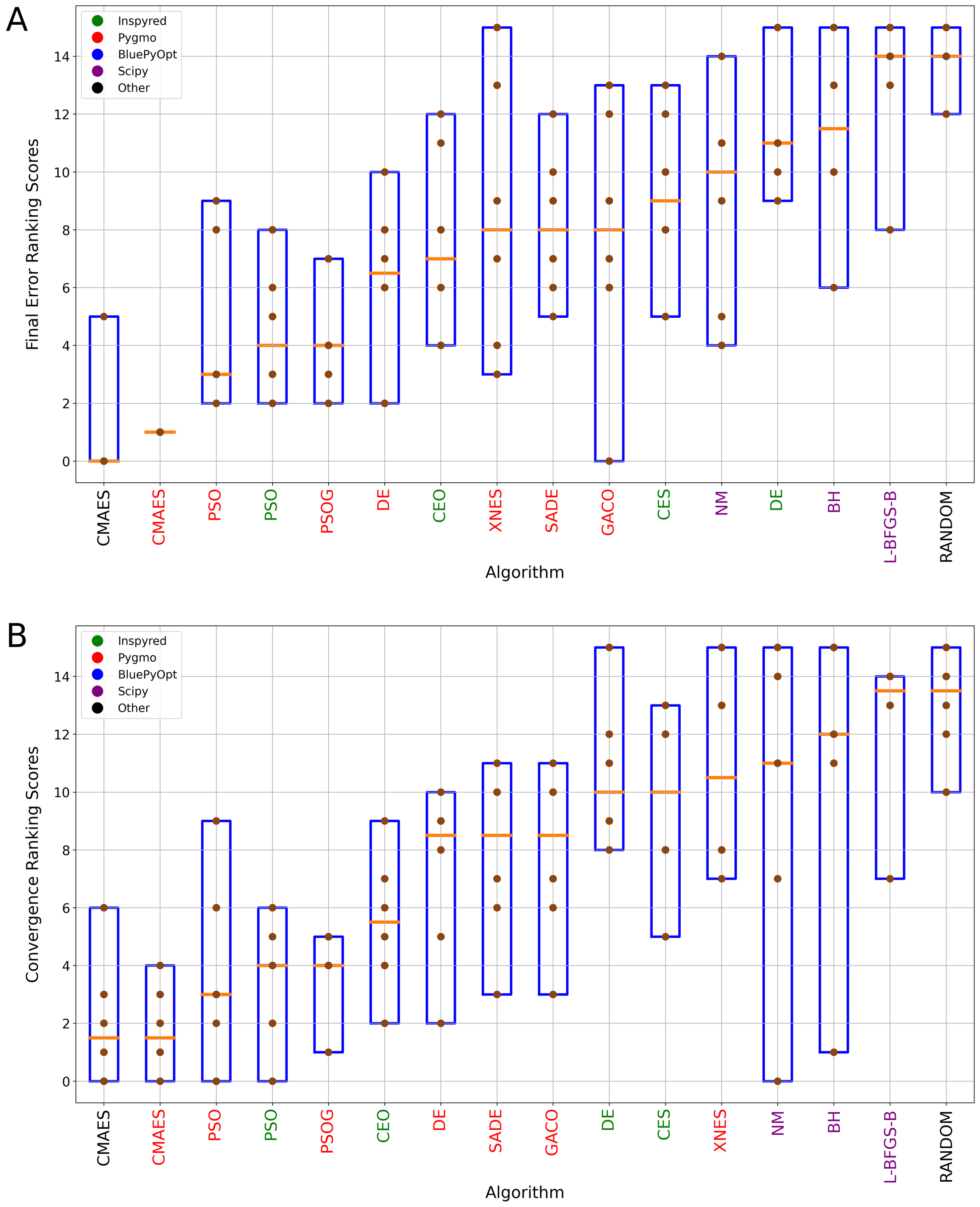

Supplement: S9 Fig — Statistics of the ranks achieved by single-objective optimization algorithms on the six different benchmarks (Figs 1–6) according to the final error (A) and convergence speed (B). Brown dots represent the ranks achieved by the algorithms in each use case; boxes indicate the full range and the orange line represents the median of these ranks. The color of the name of the algorithm indicates the implementing package, with the color code included in the legend. Algorithms are sorted according to the median of their ranks. (TIF) [file pcbi.1012039.s009.tif]
