# Supplementary material for: Evaluation and comparison of methods for neuronal parameter optimization using the Neuroptimus software framework
Source: PLoS Comput Biol. 2024 Dec 23;20(12):e1012039. doi: 10.1371/journal.pcbi.1012039 (PMC11706405; doi:10.1371/journal.pcbi.1012039)
Supplement: Attachment — Submitted filename: Response_to_reviewers_Mohacsietal2024.pdf [file pcbi.1012039.s013.pdf]

Dear Prof. Martin and Prof. Dura-Bernal,

Thank you and the three Reviewers for your detailed feedback and suggestions. In response to your comments, we have substantially revised and extended every part of the manuscript. Most importantly, to demonstrate the versatility of our framework, we added a new use case on parameter optimization in a different domain, tuning the parameters of a model of biochemical pathways involved in synaptic plasticity. We now also include additional details about our methods and results in the form of supplementary material - in particular, we have created nine new supplementary figures and two new supplementary tables (we also moved one of the existing tables from the Methods section to the supplementary material).

We have addressed all comments by the Editors and Reviewers, and provide a point-by-point response below. The original comments are shown in red font, and our responses appear in green.

We hope that our revised submission will be received favorably at the journal, and look forward to hearing from you.

Yours sincerely,

Szabolcs Káli

Response to Editors and Reviewers

Editors

After thorough evaluation by our reviewers, we have concluded that major revisions are required before your manuscript can be considered for publication. Many strengths were identified in the study, including integration of several standard open-source optimization packages, providing both an API and a GUI, offering valuable guidelines and recommendations for users, and the interactive website for sharing optimizations.

However, several key points need to be addressed, including:
1) The introduction needs more context on parameter optimization challenges, a clear research gap, and a detailed rationale for Neuroptimus.

We have extended the Introduction to state more clearly the problem that we intended to solve and the potential impact of our work.

2) The methods section lacks sufficient detail on the specific algorithms compared, the benchmark scenarios used, and the evaluation metrics.

We have extended the text of the Methods section with additional details about all aspects of our benchmarking study. In addition, we have created Supplementary Table 2, which lists all the settings and parameters for each algorithm that we used in the comparison. We also included additional details in the descriptions of the use cases, and illustrate various aspects of the experimental data in Use Case 6 in Supplementary Figures 7 and 8.

3) The results and discussion sections require additional comparisons with existing literature, a discussion on the strengths and limitations of each algorithm, and practical implications of the findings.

We have significantly extended several sections of the Discussion, especially those describing the limitations and possible extensions of our work, and also the comparisons with related work. We also added further details to the description of the results on the individual use cases.

Reviewer's Responses to Questions

Comments to the Authors:
Please note here if the review is uploaded as an attachment.

Reviewer #1:

This is a paper describing a new tool Neuroptimus, to optimize parameters of neuroscientific models. It is based on a previous tool developed previously by some of the authors. The paper also describes results obtained with the tool. Namely the comparison of different optimization algorithms to some benchmark scientific problems.

We thank Reviewer #1 for his detailed feedback on our manuscript..

I will start with a general remark I have concerning this types of papers. The authors also point it out partly in their discussion. But I'm always reluctant wrt the type of papers where creators of a software/algorithm make one-sided comparisons between code retrieved from other sources. Maybe the situation is slightly different here, since all the code they test is available as an option in their software.

We share the concerns of the Reviewer about biased comparisons. However, in this study, we are not making comparisons between our own algorithms and those developed by others. Instead, we are making available, via a unified interface, several different external software solutions developed for parameter optimization, and we aim to be as unbiased as possible in the comparisons that we provide.

The problem with search algorithms is that the very specific implementation, and especially the hyperparameters can be tremendously important for comparisons. And it will always depend on the effort of the people trying the algorithms to pick the best parameters/implementation.

So in general, and this will come back later, this should be stated as clearly as possible. I would even say in the abstract.

We agree that the results for most algorithms depend on the hyperparameters and possibly also on unspecified implementational details. We already discussed this in the Discussion, but now we have extended this section, and mention the issue of fine-tuning even in the abstract.

We would also like to add the following points. (1) We agree that many of the results could probably be improved by tuning the hyperparameters, especially if this is done separately for each use case. This can be done by changing the settings in Neuroptimus, and we actually encourage other researchers to do this and share their results (also via the Neuroptimus website). (2) On the other hand, we think it is quite valuable to know which algorithms gave consistently good results even without fine-tuning their hyperparameters (using package-default settings), as this will allow non-experts to get good results. (3) We also compare different implementations of the same algorithm to measure the impact of implementational differences.

These arguments are now also included in the manuscript.

Then some more specific comments:
Line 138: is it possible to e.g. provide constraints to the parameters. The obvious constraint is bounds, which I assume is possible. But what about other linear/nonlinear constraints?

In Neuroptimus, bound constraints can always be provided, and these are supported by all the implemented algorithms. The problem with other types of constraints is that these are not supported by many of the algorithms. One possible approach is to include these other constraints in the error function by penalizing parameter combinations that violate the constraints, and this can be implemented in Neuroptimus (although these other types of constraints currently cannot be specified through the GUI).

Wrt to this I also have general remark. Can you maybe describe how flexible your software setup is, especially wrt to the GUI. GUI's obviously make things easier for many users, but they also tend to put software in a very strict input schema. Did you e.g. take any special measures to make sure users can easily extend the GUI conceptually. (With e.g. special constraints as an example).

The GUI contains several features that increase its flexibility. This includes, among others, the possibility of using user-defined functions to describe an arbitrary mapping between abstract optimized parameters and the internal parameters of the model; the option to load arbitrary time series as inputs to the model; and, especially, the possibility of using arbitrary external scripts to run models. The wide range of use cases that we describe in the paper (including the newly added use case involving biochemical pathways) demonstrate the general flexibility of the current software. In addition, the whole software (including the GUI) is modular and relatively straightforward to extend. In fact, there are several ongoing projects that involve extending the GUI in various ways.

Line 201: Here it might be useful to cite some relevant papers that discuss surrogate data. One I can think of is Druckmann 2008

We now cite this paper in this section.

Line 241: Related to my hyperparameter comment above, did you in any way make sure that the hyperparameters of the algorithms you used were tuned for this 10k number of runs? As an example, which i'm sure you didn't do, but if e.g. one would pick a evolutionary algorithm with a 10k population size, this would obviously be bad, which is an extreme case. But many algorithms have hyperparameters that need to be tuned based on the input problem.

We decided to split the 10,000 evaluations we used as a(n arbitrary) standard as 100 generations for a population of size 100 for two reasons: (1) because it represented a reasonable balance between having large enough populations and a sufficient number of generations; and (2) because the supercomputers that we used via NSG had 128 cores per node, and thus a whole population of 100 could be evaluated in parallel on these machines.

We also did some exploratory work to change the population size of some evolutionary algorithms within reasonable limits (e.g., using 200x50 or 50x200 runs instead of 100x100), and the observed differences were minor. However, this study was not systematic enough to be included in the paper (although this remains an interesting direction to explore in future work).

Finally, our shared tools and the Neuroptimus website allow all interested researchers to run optimizations of our use cases (and others) with modified settings and compare the results to the ones that we provide. In fact, we actively encourage such extensions in the paper.

Fig 1 (and further figures). I think it would be good to (maybe in supp material?) in some way show the evolution of the subfeatures the score consists of. The total score give one view of the problem, but for a user it can be important not to just a solution with a low total score, but also a score that is low for all features at the same time as opposed to a solution with one feature being extremely low, but other still being high.

We agree that the ability to track error components during the optimization is a useful feature that was previously missing from Neuroptimus. We have now added this feature to the software so that all error components are saved individually into a JSON file for every model that is evaluated during the optimization. We demonstrate this feature in the new Supplementary Figure S1, using the results of the CMAES algorithm on the Hodgkin-Huxley use case as an example. However, we decided not to include in the paper similar results for the other algorithms and use cases, partly because this would lead to a very large number of figures (more than 100), and would also necessitate repeating all the previous optimization runs.

And related to this a more general remark. I'm not saying to redo your entire analysis, but I think

it's necessary to at least mention that the measure you use is an extremely simple one, and might not convey all the metrics that a user might be interested in. Some I can think of are: diversity of the final solution population (e.g. a user might want to build network for which a set of solutions might be required, instead of just the best one), weighing specific features and how well the algorithm follows these weights.

We have added a paragraph on other possible performance measures to the Discussion section.

It would actually be good to give a table with the capabilities of all the algorithms wrt to this. E.g. 'is' there even a population in the algorithm, is it MO, etc etc.

The manuscript includes a table (Table 2) that summarizes the main properties of each algorithm. We have also added Supplementary Table S2 that lists all the hyperparameters and other settings of the algorithms used in our benchmarking study.

And also in how far it allows parallelization. Some algorithms inherently are good to parallelize, for others it might be virtually impossible. And this can be extremely relevant wrt to speed, the 'number of evaluations necessary' is not the only metric relevant for the speed.

Table 2 also includes information about the parallelization of each algorithm and implementation. The importance of this feature is discussed extensively in the section “Algorithm recommendations based on our benchmarking results”.

Fig 2: it seems there are some missing methods wrt to the other figures, i'm not sure i saw a reason for that somewhere.

The reason why there are fewer algorithms in Figure 2 than, for instance, Figure 1, is that the Voltage Clamp use case has only a single component (mean squared error) in its error function and, therefore, only single-objective algorithms were tested and compared. This is indicated in the main text and is now also noted in the figure caption.

Line 372: Could you be a bit more specific about 'orders of magnitude' here, or what this means wrt to the actual resulting traces. Sometimes getting very close to the target trace can be perceived as overfitting the data so irrelevant. So it might be good to show what these differences mean in practice.

We agree that the actual error scores and traces carry important information, and we have therefore added additional details as supplementary information. In particular, Supplementary Figures S2-S6 show the best-fitting traces from two different algorithms (CMAES and Random Search) for Use Cases 1-5. Supplementary Table S1 contains the corresponding error

components for Use Case 4. Finally, Supplementary Figure S8 shows a detailed comparison of the feature statistics between the experimental data and the best-fitting models obtained with two different algorithms.

Fig 6A: Can you please also show the spread of the underlying data here, if necessary in supp material. It would be good to get an idea on how well these fits match with the actual raw traces.

We have added two new supplementary figures to illustrate the diversity of the experimental dataset (Supplementary Figure S7), and to provide additional information about the quality of the fits achieved by the different algorithms (Supplementary Figure S8).

Line 482: So i assume you used SO CMAES? Afaik CMA-ES also has a multi-objective variant, might be at least worth mentioning.

We now specify this explicitly in the Methods.

Line 645: One thing I'm a missing in the text is a mention that the choice of exact operators in the algorithm can be very important too, apart from hyperparameters (unless I missed it). E.g. evolutionary algorithm have a wide choice of mutation, crossover operators, elitism, etc. which can heavily influence the outcome. This is again a reason why comparing algorithms based on just names is extremely dangerous, since each algorithm name corresponds with an entire ecosystem of variants.

We now discuss this point explicitly in the Discussion section. In addition, we have added Supplementary Table S2 that lists all the settings and parameters for each algorithm that we used in the comparison.

Line 864: I'm curious if the GUI enables things like restarting from existing checkpoints etc.

Checkpointing is a very useful feature that is partially supported by Neuroptimus. The problem is that some packages and algorithms used by Neuroptimus explicitly support checkpointing, it could be added to some others with some extra programing effort, while it may not be feasible at all for some. As checkpoints are handled in a straightforward manner in BluePyOpt, we have added support for this feature to Neuroptimus (including the GUI) when it uses algorithms from BluePyOpt.

Line 993: I just wonder if it's really necessary to include all these short descriptions of these algorithms instead of pointing to paper (and maybe have a 1 sentence description in a table or so).

These descriptions were added so that readers of the paper have a concise summary of the types of algorithms supported by Neuroptimus without having to consult a large number of additional sources.

Line 1110: We don't really see these traces anywhere I think. It would be good that we at least have some links to methods that were used to generate this experimental traces.

We have added Supplementary Figure S7 that shows some of the original recordings, and also added some more details about the methods to the text.

Reviewer #2:

The authors present a new Python-based software package called Neuroptimus, that contains an API and GUI for performing neural model parameter optimization through several standard open-source optimization packages. The authors test the different optimization packages on several optimization problems of increasing complexity with the aim to provide guidelines for users with similar optimization problems. They derive several recommendations, in particular that CMAES is one of the better algorithms. The output of their optimizations are provided for the community, as well as a website for uploading additional user-defined optimizations. The interactivity through the website is an added benefit that could grow the use of the package. Most of the paper focuses around single neuron optimization. Although this has been extensively studied in the past, collating multiple libraries under Neuroptimus, may help the community leverage the experience the authors have gained through their extensive tests.

We thank Reviewer #2 for their comments on our manuscript.

One drawback of the focus on single neuron optimization is that many of the issues have been more or less solved. This would therefore limit the scope/relevance of use of their new package.

We agree with the Reviewer that there have been many successful demonstrations of single neuron parameter optimization. However, this does not mean that single neuron optimization is “solved” in the sense that no such work will be necessary in the future. On the contrary: single neuron optimization is becoming a standard tool, both in the investigation of single neuron functions and as an essential step in the construction of detailed network models. This means that many labs that are not experts on parameter optimization would like to perform single neuron optimization, ideally without having to learn all the technical details. One important goal of our software development was to provide such an intuitive tool for parameter optimization to non-expert researchers.

Furthermore, Neuroptimus is by no means limited to the optimization of biophysical models of single neurons. Through creative use of its many features (such as user-defined functions and external simulators), it supports the optimization of almost any type of model based on any kind of quantitative data.

For that reason, I would like to see some additional studies (though certainly not with the same depth/breadth as for single neuron optimization) on neuronal network optimization and sub-cellular optimization (e.g. RxD).
The authors mention that they have already performed these specific studies in the Discussion. Including the details and results of those studies would increase the appeal of their work and allow a wider audience to benefit. In addition, it would showcase how to use their software for computational optimization tasks that are less "solved", or generally solvable with more effort than single neuron optimization which has a broad literature spanning decades.

We agree with the Reviewer that showing use cases outside the domain of single cell biophysical models would demonstrate the generality of our framework and may increase the impact of our work. Therefore, we now include an example from a different domain: we fit the parameters (initial concentrations and input fluxes) of a detailed model of the main biochemical pathways involved in long-term synaptic plasticity in a dendritic spine, in order to capture the changes in synaptic efficacy in an experiment where long-term potentiation was induced. The results are described in the new section “Beyond single-cell biophysical modeling: optimizing a model of biochemical pathways related to long-term synaptic plasticity” and Figure 8, while the technical details of this new use case are summarized in the section “Use Case 7: Intracellular biochemical pathway model” of the Methods.

On the other hand, our work on the optimization of network parameters (such as synaptic weights) is not ready to be shared yet, and will be described in a future paper. Therefore, we have removed the reference to existing results in this domain, and mention it only as a possible future direction.

Towards this end of network optimization, I would suggest highlighting several recent papers that used evolutionary strategies to fit model parameters to reproduce experimental data:
Data-driven multiscale model of macaque auditory thalamocortical circuits reproduces in vivo dynamics

or to teach models to perform behaviors through Evolutionary Strategy:
Training spiking neuronal networks to perform motor control using reinforcement and evolutionary learning

These approaches could form the basis of comparison for a network based approach to optimization through Neuroptimus.

We thank the Reviewer for pointing out these recent and relevant papers, which have been added to our manuscript.

Detailed comments

abstract:
"Recently, manual model tuning has been replaced by automated parameter search using a variety of different tools and methods."
"Recently" - automated parameter optimization has been used for decades

While it is true that automated methods have been used for a long time in computational neuroscience, until recently most models were still developed via manual tuning. Although we did not quantify this systematically, our impression is that automated tuning became dominant only in the last few years. We have changed the wording in the abstract to make this clearer.

Introduction:
Left out an important, recent paper on optimizing single neuron models to experimental data using
evolutionary multiobjective optimization:
Journal of Neurophysiology: 117(1):148-162, 2017

We thank the Reviewer for highlighting this publication, which is now included and discussed in our manuscript (please see below for further details).

The study is well done and interesting but the authors claim in several places that the most widely performed optimization in neuroscience is on the single neuron level. This may have been true in the past, but lately, a lot of recent state-of-the-art work has been done on optimizing detailed neuronal circuit models to replicate the patterns seen in electrophysiological data, by several different groups:
Data-driven multiscale model of macaque auditory thalamocortical circuits reproduces in vivo dynamics

Multiscale model of primary motor cortex circuits predicts in vivo cell-type-specific, behavioral state-dependent dynamics

Reconstructing visual experience from a large-scale biologically realistic model of mouse primary visual cortex

Uncovering circuit mechanisms of current sinks and sources with biophysical simulations of primary visual cortex

HNN-core: A Python software for cellular and circuit-level interpretation of human MEG/EEG

We thank the Reviewer for drawing our attention to these recent papers. We now cite several of them in the manuscript.

Although it may be beyond the scope of the current work to include network/circuit-based optimization,
not even discussing this latest trend on a paper on neuronal optimization, seems to be missing an important part of current reseearch. Can the authors include more discussion of network optimization?

We now discuss network optimization more extensively based on the papers suggested by the Reviewer as well as others, including our own work on modeling hippocampal network dynamics, representations and learning (Ecker et al., 2022).

Alternatively, it would be useful if they can extend their software to include network optimization on a simple data-set. At least this would broaden the audience for their software and keep it relevant.

Optimizing network parameters is feasible in the current version of Neuroptimus, and we considered adding an example of this to the paper. However, due to the amount of extra work this would have involved, we decided that this was beyond the scope of the current revision, and will be addressed in future work.

Previous work mentioned above (Neymotin 2017) uses a step-wise approach to optimizing conductance-based neuronal models.
The first stage involves fitting the passive parameters (leak conductance, capacitance, etc.) to subthreshold
current injections. Afterwards active channel conductances contributing to spike generation are fitted
to reproduce accurate firing patterns (rate vs injected current at different steps, action potential shape, etc.). In that study, the authors found better performance using this strategy. Can the authors
provide a similar step-wise comparison instead of optimizing all functions at once? Or if this is not possible in general, can they comment on effective strategies for dealing with these types of complexities?

Such multi-step approaches are feasible with Neuroptimus, but they currently require some manual adjustments in the settings between the steps. Extending the software to handle such workflows automatically is an interesting future direction. We now discuss the paper by Neymotin et al. and the related capabilities of Neuroptimus in the manuscript.

Discussion line 554: the authors mention only NEURON supports a GUI. That is not correct, as far as I know NetPyNE and Human Neocortical Neurosolver both have GUIS and both support optimization of neuronal network models.

We thank the Reviewer for these suggestions. We have looked at the documentation of these tools as well as the published examples of research that used them. As far as we could tell, the NetPyNE GUI supports only grid search, although other methods can be used programmatically.

Similarly, the HNN GUI appears to support only a single local optimization method (COBYLA), although other methods are available when using HNN-Core. In addition, HNN is not a general-purpose optimization tool (it is based on a specific model of neocortical circuitry). Nevertheless, the Discussion section has been updated to make our claims completely accurate.

If CMAES and PSO are the best algorithms, why include all the others? Were any of the others best in certain circumstances?

Yes, some other algorithms performed very well in certain use cases (e.g., GACO returned the best final results for the detailed compartmental model of a CA1 pyramidal cell, and local algorithms such as Nelder-Mead were the fastest to find the best solution when passive parameters were tuned in Use Case 3). In addition, the performance of most algorithms depends on their hyperparameters, and it is likely that their performance could be improved by changing these settings, especially if these are adjusted for individual use cases. Including many algorithms and allowing the user to set their hyperparameters should immediately benefit expert users, and also allow all users to benefit from new results and suggested good settings for any of the included algorithms. We have extended the Discussion to include some of these points.

While claiming that many algorithms are a benefit to the users, naive users will not know which algorithm to select. Perhaps having clear default algorithms and parameters in the GUI and code-base would make it easier for the novice user to find the algorithmic equivalent of the "needle in the haystack". Can the authors comment on this tradeoff between large number of algorithms vs best algorithms and how users might be encouraged to use the best algorithms by default?

The Neuroptimus GUI contains a list of recommended algorithms in the algorithm selection part of the GUI. This fact is now highlighted in the manuscript.

Possible Extensions in Discussion is an interesting section that mentions use of Neuroptimus outside the scope of single cell neuronal modeling. I would like to see some of the results of the the NEURON RxD optimization simulation and the network model in particular, since it broadens the scope of the software for more interesting/timely research questions, even if not including a comparison of all optimization algorithms. Without these examples, the tool may not be as attractive to the research community. Note that the optimizations and network models describe above could have benefitted from a tool such as the one described in the manuscript. By only focusing on single cell models, the authors detract from potential users of the Software.

We have included a new example based on a subcellular biochemical pathway modeled in the rxd module of Neuron (see above).

Methods

The authors mention models should generally be implemented in HOC. HOC is a bit outdated, and most new NEURON models are specified in Python. Can the authors clarify if Python implementations of neuronal models are supported?

Previously, Neuroptimus only supported Neuron models implemented in HOC. However, prompted by this comment of the Reviewer, we have extended Neuroptimus so that it is now able to handle Neuron models both in HOC and Python. We have also modified the manuscript accordingly.

Reviewer #3:

We thank Reviewer #3 for their comments and suggestions.

1. The introduction sets the stage for the study but could be strengthened by:
Providing more context on the challenges of parameter optimization in computational neuroscience.
Clearly stating the research gap or problem that the study aims to address.
Including a more detailed rationale for the development of the Neuroptimus software framework and its potential impact on the field.

We have extended the Introduction to state more clearly the problem that we intended to solve and the potential impact of our work.

2. Methods: The methods section lacks sufficient detail on the specific algorithms compared, the benchmark scenarios used, and the parameters for optimization. To enhance the methods section, the authors could consider providing a clear description of each algorithm tested, including any modifications or adaptations made. The authors should also detail the benchmark scenarios used, explaining their relevance to neuronal parameter optimization and clearly outline the evaluation metrics used to compare the performance of the algorithms.

We have extended the text of the Methods section with additional details about all aspects of our benchmarking study. In addition, we have created Supplementary Table 2, which lists all the settings and parameters for each algorithm that we used in the comparison. We also included additional details in the descriptions of the use cases, and illustrate various aspects of the experimental data in Use Case 6 in Supplementary Figures 7 and 8.

3. Results: In lines 1066-1084, the authors describe some dendritic features and channel distributions. However, the authors should describe how Neuroptimus fares with extracting dendritic parameters such as the attenuation of back-propagating action potentials and PSP amplitudes as a function of dendritic section distance from the soma.

Neuroptimus supports using dendritic voltage traces for the extraction of features and the calculation of errors. However, this does not fully cover many important use cases involving AP back-propagation, PSP attenuation, or synaptic integration. However, we note that we currently have a project that aims to extend Neuroptimus so that it will be able to use the results of unit tests from our validation package HippoUnit (based on the SciUnit framework). This would allow optimization based on essentially any combination of (quantitative) neuronal properties.

4. Discussion: Suggestions for improvement in the discussion section include:
Discussing the strengths and limitations of each algorithm tested to the benchmark scenarios.
Comparing the findings with existing literature on neuronal parameter optimization methods.
Highlight the practical implications of the results for researchers in computational neuroscience.

We have significantly extended several sections of the Discussion, especially those describing the limitations and possible extensions of our work, and also the comparisons with related work. We also added further details to the description of the results on the individual use cases.
